# Developmental Exposure to Polychlorinated Biphenyls Interferes with Experience-Dependent Dendritic Plasticity and Ryanodine Receptor Expression in Weanling Rats

**DOI:** 10.1289/ehp.11771

**Published:** 2008-09-12

**Authors:** Dongren Yang, Kyung Ho Kim, Andrew Phimister, Adam D. Bachstetter, Thomas R. Ward, Robert W. Stackman, Ronald F. Mervis, Amy B. Wisniewski, Sabra L. Klein, Prasada Rao S. Kodavanti, Kim A. Anderson, Gary Wayman, Isaac N. Pessah, Pamela J. Lein

**Affiliations:** 1 Center for Research on Occupational and Environmental Toxicology, Oregon Health & Science University, Portland, Oregon, USA;; 2 Veterinary Molecular Biosciences and Center for Children’s Environmental Health, University of California, Davis, California, USA;; 3 Neurostructural Research Labs and Center for Aging and Brain Repair, University of South Florida College of Medicine, Tampa, Florida, USA;; 4 Neurotoxicology Division, National Health and Environmental Effects Research Laboratory, U.S. Environmental Protection Agency, Research Triangle Park, North Carolina, USA;; 5 Department of Psychology, Florida Atlantic University, Boca Raton, Florida, USA;; 6 Department of Pediatrics, University of Oklahoma Health Sciences Center, Oklahoma City, Oklahoma, USA;; 7 Department of Molecular Microbiology and Immunology, Johns Hopkins Bloomberg School of Public Health, Baltimore, Maryland, USA;; 8 Department of Environmental & Molecular Toxicology, Oregon State University, Corvallis, Oregon; USA;; 9 Department of Veterinary and Comparative Anatomy, Physiology and Pharmacology, College of Veterinary Medicine, Washington State University, Pullman, Washington, USA;; 10 Department of Environmental Health Science, Johns Hopkins Bloomberg School of Public Health, Baltimore, Maryland, USA

**Keywords:** dendrite, neurodevelopmental disorders, developmental neurotoxicity, PCBs, plasticity, ryanodine receptor

## Abstract

**Background:**

Neurodevelopmental disorders are associated with altered patterns of neuronal connectivity. A critical determinant of neuronal connectivity is the dendritic morphology of individual neurons, which is shaped by experience. The identification of environmental exposures that interfere with dendritic growth and plasticity may, therefore, provide insight into environmental risk factors for neurodevelopmental disorders.

**Objective:**

We tested the hypothesis that polychlorinated biphenyls (PCBs) alter dendritic growth and/or plasticity by promoting the activity of ryanodine receptors (RyRs).

**Methods and Results:**

The Morris water maze was used to induce experience-dependent neural plasticity in weanling rats exposed to either vehicle or Aroclor 1254 (A1254) in the maternal diet throughout gestation and lactation. Developmental A1254 exposure promoted dendritic growth in cerebellar Purkinje cells and neocortical pyramidal neurons among untrained animals but attenuated or reversed experience-dependent dendritic growth among maze-trained littermates. These structural changes coincided with subtle deficits in spatial learning and memory, increased [^3^H]-ryanodine binding sites and RyR expression in the cerebellum of untrained animals, and inhibition of training-induced RyR upregulation. A congener with potent RyR activity, PCB95, but not a congener with negligible RyR activity, PCB66, promoted dendritic growth in primary cortical neuron cultures and this effect was blocked by pharmacologic antagonism of RyR activity.

**Conclusions:**

Developmental exposure to PCBs interferes with normal patterns of dendritic growth and plasticity, and these effects may be linked to changes in RyR expression and function. These findings identify PCBs as candidate environmental risk factors for neurodevelopmental disorders, especially in children with heritable deficits in calcium signaling.

Polychlorinated biphenyls (PCBs) are a structurally related group of stable and highly lipophilic chemicals with widespread distribution in the environment (Hornbuckle et al. 2006). Despite being banned in 1977, PCBs persist in the environment, and high residue levels are still detected in human tissues (DeCaprio et al. 2005; Humphrey et al. 2000; Park et al. 2007). Epidemiologic data indicate that PCBs negatively impact neuropsychologic function in exposed children (Carpenter 2006; Korrick and Sagiv 2008; Schantz et al. 2003), and experimental animal studies confirm that developmental PCB exposure causes cognitive and psycho-motor deficits (Mariussen and Fonnum 2006). However, the cellular and molecular mechanisms mediating these effects remain speculative.

PCBs interfere with endocrine functions, specifically those mediated by thyroid hormone (Zoeller 2007) and estrogen (DeCastro et al. 2006; Dickerson and Gore 2007), and increase neuronal Ca^2+^ levels via several mechanisms (Kodavanti 2005; Mariussen and Fonnum 2006), including ryanodine receptor (RyR) activation (Pessah and Wong 2001). It is not clear, however, how these molecular effects relate to PCB developmental neurotoxicity. Thyroid hormone (Kapfhammer 2004), estrogen (Cooke and Woolley 2005), and Ca^2+^ (Lohmann and Wong 2005; Redmond and Ghosh 2005) influence neuronal connectivity via dynamic control of dendritic structure. Altered patterns of dendritic growth and plasticity are associated with impaired behavior in experimental models (Berger-Sweeney and Hohmann 1997) and are thought to contribute to diverse neurodevelopmental disorders (Connors et al. 2008; Pardo and Eberhart 2007; Zoghbi 2003), suggesting the possibility that PCBs elicit developmental neurotoxic effects by interfering with neuronal connectivity.

Consistent with this hypothesis, we recently observed that developmental PCB exposure disrupts the balance of neuronal inhibition to excitation in the developing rat auditory cortex (Kenet et al. 2007) and accelerates dendritic growth in the hippocampus and cerebellum of weanling rats (Lein et al. 2007). Questions yet to be addressed include the relationship between PCB interference with neuronal connectivity and known molecular targets of PCBs and whether developmental exposures to PCBs interfere with experience-dependent dendritic plasticity, a phenomenon critical to associative learning and memory (Leuner and Shors 2004). In this study, we used the Morris water maze as a tool for inducing experience-dependent neural plasticity in weanling rats exposed to Aroclor 1254 (A1254) in the maternal diet throughout gestation and lactation. In addition to assessing dendritic morphology in these animals, we also quantified RyR expression and function, thyroid hormone levels, and developmental end points regulated by sex steroids. Our findings suggest that developmental PCB exposure interferes with dendritic growth and plasticity coincident with delayed spatial learning and that perturbation of RyR expression and function contributes to these effects.

## Materials and Methods

Detailed descriptions of all methods are available online in Supplemental Material (http://www.ehponline.org/members/2008/11771/suppl.pdf).

### Animals and PCB exposures

Animals were treated humanely and with regard for alleviation of suffering according to protocols approved by the Institutional Animal Care and Use Committees of the Johns Hopkins University and Oregon Health & Science University. Adult Long Evans rats were purchased from Charles River Laboratories (Hollister, CA) and housed individually, except during breeding, in standard plastic cages with Alpha-Dri bedding (Shepherd Specialty Papers, Watertown, TN) in a temperature-controlled (22 ± 2°) room on a 12-hr reverse light**–**dark cycle. Food and water were provided *ad libitum*. Dams used in the study delivered litters of 10–15 pups (*n* = 11 dams per treatment group). By postnatal day 2 (P2), litters were culled to five males and five females. Pups were weaned on P21.

Dams were dosed with the commercial PCB mixture Aroclor 1254 (A1254, lot #124-191; AccuStandard, New Haven, CT) at 1 mg or 6 mg/kg/day beginning 2 weeks prior to breeding and continuing until P21. A1254 was diluted in corn oil and pipetted onto one-half of a Keebler Golden Vanilla Wafer (Kellogg Company, Battle Creek, MI). Control animals received wafers dosed with an equal volume (500 μL) of vehicle. Doses were adjusted daily to account for changes in body weight of the dams. Dams were fed the wafers in a separate cage to prevent the pups from accessing the wafers and were watched carefully to ensure that the entire wafer was consumed (typically within 5 min).

### Tissue culture and transfection

High-density cultures of dissociated neocortical neurons (10^5^ cells/cm^2^) were prepared from P1 Sprague Dawley rats (Charles River Laboratories) and maintained in Neurobasal-A (Invitrogen, Carlsbad, CA) supplemented with B27 (Invitrogen) as previously described (Wayman et al. 2006). On day 6 *in vitro* (6-DIV) cultures were transfected with plasmid encoding a microtubule-associated protein-2 (MAP2)-enhanced green fluorescent protein (GFP) fusion construct (Wayman et al. 2006) using Lipofectamine-2000 (Invitrogen) according to the manufacturer’s protocol. On 7-DIV, cultures were treated for 48 hr with vehicle (DMSO at 1:1,000 dilution), PCB95 (2,2′,3,5′,6-pentachlorobiphenyl, > 95% purity; AccuStandard), or PCB66 (2,3′,4,4′-tetrachlorobiphenyl, > 95% purity; AccuStandard).

### Thyroid hormone assays

Total thyroxine (T_4_) and triiodothyronine (T_3_) levels were determined in serum samples by radioimmunoassay (Diagnostic Products Corp, Los Angeles, CA) as previously described (Kodavanti et al. 1998).

### Analysis of reproductive development

At P2, litter size, sex ratio, and pup body mass were measured. Anogenital distance (AGD) was measured at P2, P10, and P21. At P21, litters were weaned and housed with same-sex siblings. At P40 (puberty), body mass was measured, and the presence of preputial separation or vaginal opening was recorded in males and females, respectively. At P70, blood samples collected from the retro-orbital sinus were analyzed for serum levels of testosterone in males and estradiol in females by radioimmunoassay per the manufacturer’s protocol [ICN Biochemicals, Inc. (MP Biomedical, Solon, OH)]. Samples were assayed in triplicate and cross-reactivity with other steroids was < 0.1%. After blood collection, animals were euthanized, and reproductive organs were removed and weighed. The tunica was then stripped from paired testes, and the seminiferous tubules were homogenized in 0.5% Triton-X 100 with 0.01% thimerosal to determine sperm concentration using a Newbauer chamber.

### Morris water maze

Spatial learning and memory was assessed on P24 in one male and one female from 11 different litters within each treatment group, using the Morris water maze as previously described (Jett et al. 1997). Rats were tested in one trial per day, except on the first day, when two trials were administered. This modification increases difficulty such that relatively small differences between treatment groups can be detected, yet the task is not too difficult for rats to learn quickly (Jett et al. 1997, 2001; Kuhlmann et al. 1997). An escape latency of 10 sec was chosen as the criterion that animals had learned the task, based on previous studies using rats of comparable age in a similar size pool (Jett et al. 1997, 2001; Markwiese et al. 1998; Rudy et al. 1987). To test spatial memory, a probe test was administered 30 min after the spatial training trials on the first day that the mean escape latency of any treatment group reached criterion.

### Morphometric analyses

On P31, animals were euthanized and perfused with 4% paraformaldehyde. To visualize Purkinje cell dendritic arbors, parasagittal cryosections (12 μm) were cut from both cerebellar hemispheres, starting 1 mm from the midline, and reacted with antibody specific for calbindin-D_28K_ (Sigma, St. Louis, MO), which specifically labels Purkinje cells (Christakos et al. 1987). Dendritic arbors of neocortical neurons were visualized by Golgi staining as previously described (Lein et al. 2007). Dendritic arbors were quantified in cultured neocortical neurons transfected with MAP2-eGFP as previously described (Wayman et al. 2006). An average of 10 neurons per culture from three cultures per group was analyzed, and results were confirmed in two independent dissections.

### RyR profiling

Specific [^3^H]-ryanodine (5 nM) binding to whole particulate cerebellar membranes was measured at P21 and P31, as previously described (Wong et al. 1997). Western blot analyses were used to quantify RyR expression as previously described (Roegge et al. 2006).

### Cytochrome-P450 (CYP) activity

Hepatic CYP content was determined as previously described (Omura and Sato 1964). 7-Ethyoxyresorufin *O*-deethylase (EROD) and 7-pentoxyresorufin *O*-depentylase (PROD) activities were analyzed in hepatic microsomes according to the method of Lubet (Lubet et al. 1990) as modified by Kennedy and Kono (Kennedy and Jones 1994; Kono et al. 1999). Enzyme activities were normalized to protein concentration as determined using the BCA Protein Assay (Pierce, Rockford, IL).

### Congener-specific PCB analyses

Whole brains from P31 rats were stored at –80°C and thawed immediately before extraction, cleanup, and fractionation using gel permeation chromatography as previously described (Sethajintanin et al. 2004).

## Results

### Developmental A1254 exposure did not cause maternal or fetal toxicity

**Figure 1:**
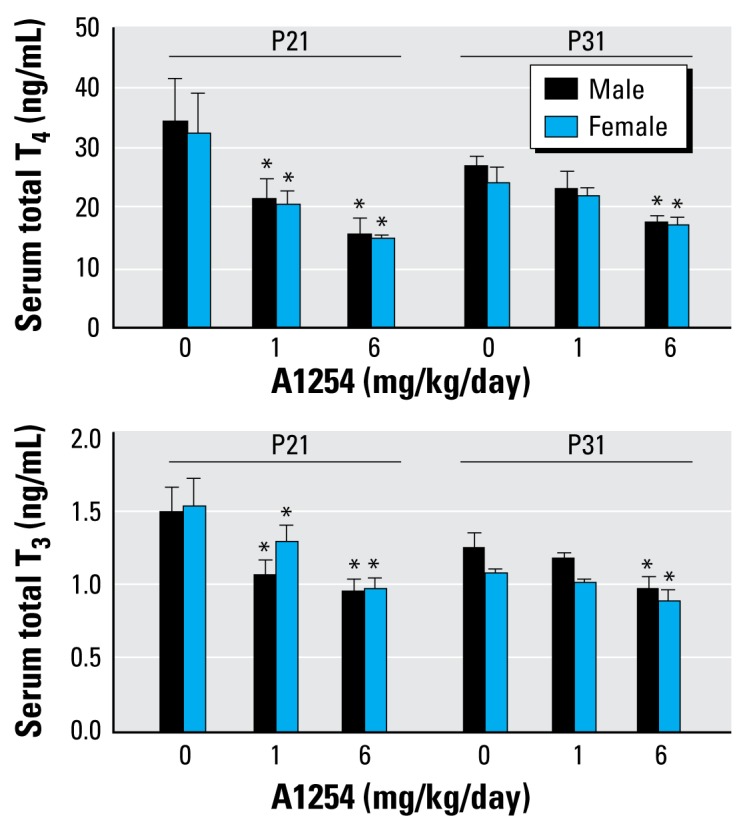
Developmental A1254 exposure decreases serum thyroid hormone levels. Developmental A1254 exposure caused significant dose- dependent decreases in total serum T_4_ and T_3_ at P21. This effect persisted in the 6 mg but not the 1 mg/kg/day A1254 treatment group at P31. No sex differences were observed. Data are presented as mean ± SEM (*n* = 7–9/group). *p < 0.05 (two-way ANOVA, with treatment and sex as main effects; Fisher’s LSD post hoc).

Consistent with previous reports (Roegge et al. 2004), dietary exposure of dams to A1254 at 1 mg or 6 mg/kg/day, starting 2 weeks before conception and continuing throughout gestation and lactation, did not negatively impact developmental outcomes or cause overt signs of intoxication in dams or pups as determined by lack of treatment-related changes in maternal weight gain during gestation, maternal body weight during lactation, length of gestation, litter size, and weight gain of offspring during lactation [see Supplemental Material (http://www.ehponline.org/members/2008/11771/suppl.pdf), Figure 1].

### Effects of developmental A1254 exposure on thyroid hormone levels and sex steroid-dependent developmental end points

Developmental exposure to A1254 at 1 mg or 6 mg/kg/day significantly decreased serum concentrations of T_4_ and T_3_ at P21 (Figure 1). By P31, serum T_3_ and T_4_ had recovered to control levels among pups in the 1 mg/kg/day A1254 treatment group, but were still significantly depressed in the 6 mg/kg/day treatment group. No sex differences were observed.

As shown in Table 1, developmental A1254 exposure differentially altered a subset of reproductive developmental end points regulated by estrogens and androgens including: *a*) male to female ratio of litters, which was increased in the 6 mg/kg/day A1254 treatment group; *b*) absolute but not relative AGD among female offspring, which was longer than control in both A1254 treatment groups; *c*) vaginal opening, which was significantly delayed among females in the 6 mg/kg/day A1254 treatment group; and *d*) both absolute and relative prostate mass, which was increased in P70 males exposed to A1254 at 1 mg/kg/day but not 6 mg/kg/day, in the maternal diet. A number of other sex steroid–dependent developmental end points were not affected by developmental exposure to either dose of A1254, including AGD (absolute or relative) in males, preputial separation in males, the size of female reproductive organs, the size of male reproductive organs other than the prostate, sperm counts or plasma concentrations of estradiol in females and testosterone in males.

### Developmental A1254 exposure alters spatial learning and memory

The Morris water maze has been shown to detect subtle but significant changes in cognitive function in weanling rats exposed to developmental neurotoxicants (Jett et al. 1997, 2001; Markwiese et al. 1998), and training in this task induces experience-dependent dendritic growth (Greenough et al. 1979). Although considered a test of hippocampal function, performance in the Morris water maze is also dependent on the function of the cerebellum and neocortex (Lalonde and Strazielle 2003; Save and Poucet 2000), which is particularly relevant to the current studies because *a*) the vulnerability of the cerebellum to developmental hypothyroidism (Dong et al. 2005) is proposed as a primary mechanism for PCB developmental neurotoxicity (Koibuchi and Iwasaki 2006; Roman 2007; Zoeller 2007); and *b*) our previous studies of molecular bio-markers of dendritic growth (Lein et al. 2007) and development of excitatory to inhibitory balance in neurotransmission (Kenet et al. 2007) indicated that neuronal connectivity in the neocortex may be particularly susceptible to modulation by PCBs.

**Figure 2:**
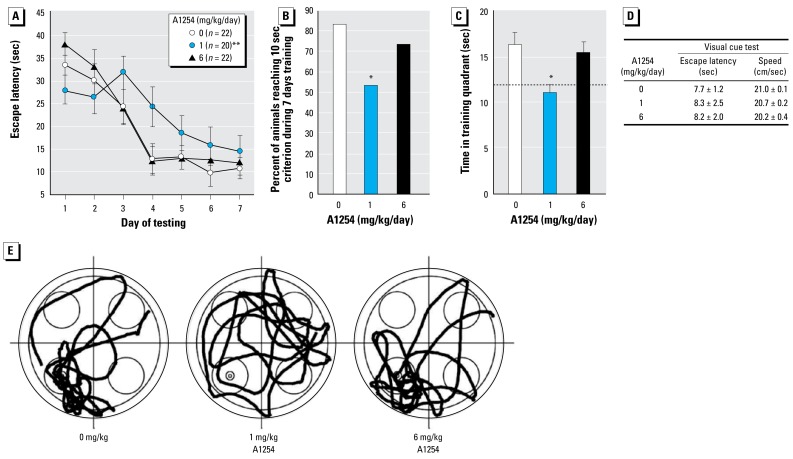
Developmental A1254 exposure at 1 mg but not 6 mg/kg/day impairs performance in the Morris water maze. (*A*) Escape latency as a function of trial. (*B* ) Percentage of animals that reached criterion (escape latency ≤ 10 sec) during the 7-day training period. (*C*) Probe tests conducted on day 4. Dotted line indicates predicted time spent in the training quadrant by chance alone. (*D*) Performance in visual cue test and swimming speed. (*E*) Representative swim paths from probe tests on day 4. Data are presented as mean ± SEM. *p< 0.05, **p < 0.01 ( *A*, repeated measures ANOVA; *B*, Fisher’s exact test; *C* and *D*, ANOVA; Newman-Keuls post hoc).

Training in the Morris water maze was initiated on P24 and concluded on P30 (Figure 2). Repeated-measures two-way ANOVA, with sex and treatment as the between-subjects factors and trial day as the repeated-measures factor, identified a significant interaction between treatment and trial (*p* < 0.001) but a lack of interaction of sex with treatment or trial. There was no three-way interaction among trial, treatment, and sex. Subsequent post hoc Newman-Keuls multiple comparisons on each trial day revealed a significant difference in escape latency between the 1 mg/kg/day treatment group and both vehicle and 6 mg/kg/day treatment groups on day 4. Although significant differences between treatment groups were not detected on other trial days, it is clear from the plot of escape latency that from day 3 through day 7, the 1 mg/kg/day treatment group took longer to find the platform than the control and 6 mg/kg/day treatment group (Figure 2A). The percentage of animals within each treatment group that reached criterion by the end of the training period was significantly reduced in the 1 mg but not 6 mg/kg/day treatment group relative to controls (Figure 2B), further suggesting that weanling rats exposed developmentally to A1254 at 1 mg/kg/day were not as proficient at spatial learning as animals in either the control or 6 mg/kg/day A1254 group. Spatial memory was assessed during the probe test on days 4 and 7, the first day that controls and 1 mg/kg/day–treated animals reached criterion, respectively. On day 4, the 1 mg/kg A1254 rats spent significantly less time in the training quadrant than those from the control or 6 mg/kg/day A1254 groups (Figures 2C and 2E). On day 7, however, there were no significant differences between groups (data not shown), suggesting that although learning and memory were impaired in the 1 mg/kg/day A1254 group, with additional training, rats in this group did acquire the task. Developmental exposure to A1254 at either dose had no effect on escape latency in the visual cue test or on swimming speed (Figure 2D), indicating that learning and memory deficits observed in the training trials and probe tests were not due to negative impacts of A1254 on vision, motivation, or swim speed.

### Developmental A1254 exposure interferes with dendritic growth and plasticity

Morphometric analyses of Nissl-stained sections indicated no overt treatment-related effects on development of the cerebellum or neocortex [see Supplemental Material (http://www.ehponline.org/members/2008/11771/suppl.pdf), Figure 2]. To assess effects of developmental A1254 exposure on cellular indices of neural circuitry, dendritic length was quantified in individual cerebellar Purkinje cells and neocortical pyramidal neurons at P31. Because no sex differences were observed in PCB effects on performance in the Morris water maze, morphometric studies were restricted to males.

**Figure 3:**
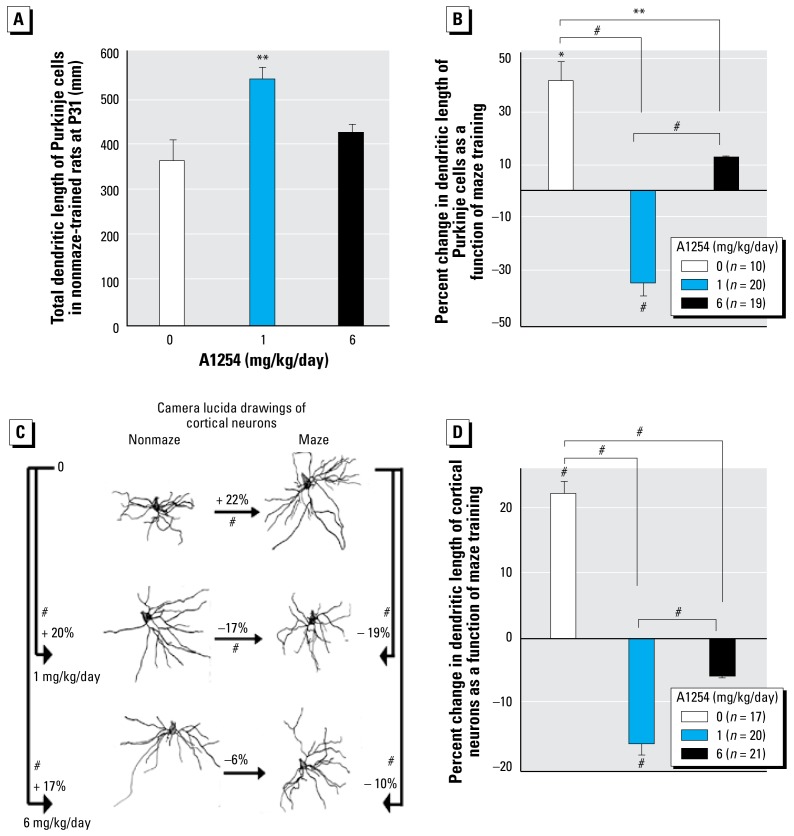
Developmental A1254 exposure interferes with normal dendritic growth and experience-dependent dendritic plasticity. Dendritic morphology was analyzed among P31 rats trained in the Morris water maze (Maze) and among littermates identically housed and exposed but not trained (Nonmaze). (*A*) Total dendritic length of cerebellar Purkinje cells in nonmaze-trained animals. (*B*) Significant effect of maze training on total dendritic length of Purkinje cells. (*C*, *D*) Effects of maze training and developmental A1254 exposure on dendritic growth in cortical neurons. Data are presented as mean ± SEM (*n* = 17–21 neurons/group). Percent change in dendritic length as a function of maze training was calculated as the difference in dendritic length of neurons in maze-trained animals versus nonmaze-trained animals divided by dendritic length of neurons in maze-trained animals multiplied by 100. *p < 0.05, **p < 0.01, #*p* < 0.001 (*A* and *B*, ANOVA followed by Newman-Keuls; *C* and *D*, Wilcoxon test).

Morphometric analyses of cerebellar neurons immunopositive for calbindin, which is a specific marker of Purkinje cells (Christakos et al. 1987), indicated that among untrained animals, developmental exposure to A1254 at 1 mg but not 6 mg/kg/day significantly increased total dendritic length relative to vehicle controls (Figure 3A). Analysis of the percent change in dendritic length of cerebellar Purkinje cells as a function of maze training within groups revealed that Morris water maze training significantly increased total dendritic length in Purkinje cells of controls, caused significant dendritic retraction in the 1 mg/kg/day A1254 group, and had no significant effect on dendritic length among rats in the 6 mg/kg/day A1254 group (Figure 3B). Comparison between groups indicated that training-induced dendritic growth observed among controls was significantly attenuated by developmental exposure to A1254 at 6 mg/kg/day and actually reversed by A1254 at 1 mg/kg/day (Figure 3B).

Representative camera lucida drawings of the basilar dendritic arbor of neocortical pyramidal neurons from untrained and maze-trained littermates within each group (Figure 3C) demonstrate effects similar to those observed in cerebellar Purkinje cells. Quantification of dendritic length by Sholl analysis indicated that in untrained animals, developmental exposure to A1254 increased dendritic length in neocortical pyramidal neurons by 20% and 17% in the 1 mg and 6 mg/kg/day groups, respectively, relative to controls. Maze training increased dendritic length of neocortical neurons among controls by 22% but caused dendritic length to decrease by 17% in the 1 mg/kg/day A1254 treatment group (Figures 3C and 3D). In animals exposed developmentally to A1254 at 6 mg/kg/day, maze training caused neither significant expansion nor retraction of the dendritic arbor relative to untrained littermates (Figures 3C and 3D). Comparison between groups of the percent change in dendritic length of neocortical pyramidal neurons as a function of maze training indicated that the training-induced dendritic growth observed in controls was inhibited by developmental exposure to A1254, with significantly more pronounced effects observed in the 1 mg versus 6 mg/kg/day A1254 group (Figure 3D).

### Developmental A1254 exposure influences RyR profiles

**Figure 4:**
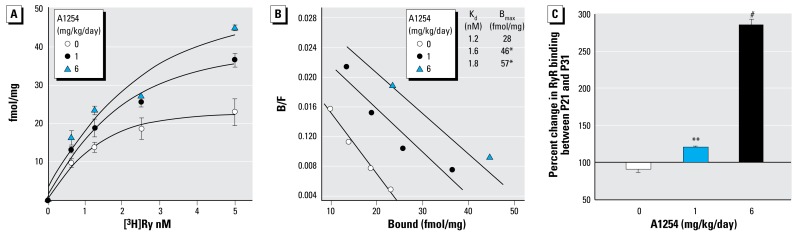
Developmental A1254 exposure increases specific [^3^H]-ryanodine (Ry) binding to cerebellar membranes. Binding constants (*K*_d_ and *B*_max_) were determined from a [^3^H]-ryanodine–binding curve measured in cerebellar membranes from P21 pups (*A*) using Scatchard analysis (*B*). (*C*) Measurements of specific binding of [^3^H]-ryanodine (5 nM) to cerebellar membranes from P21 and maze-trained P31 rats. The percent change in ryanodine binding between P21 and P31 was calculated as the difference in B_max_ between P21 and P31 divided by B_max_ at P21 multiplied by 100. Data are presented as mean ± SD (*n* = 4). *p< 0.05, **p < 0.01, #*p* < 0.001 (ANOVA; Newman-Keuls post hoc).

One of the most sensitive molecular targets of PCBs is RyR activation (Pessah and Wong 2001). All three RyR isoforms are expressed in the brain, and RyR activity influences use-dependent synaptic plasticity (Berridge 2006). These observations suggest that PCBs may interfere with dendritic growth and plasticity via RyR-mediated mechanisms. To test this hypothesis, we first determined whether developmental A1254 exposure influenced RyR function and expression. Because the effects of developmental A1254 exposure on dendritic growth and plasticity were similar between cerebellar Purkinje cells and neocortical pyramidal neurons, these studies focused on the cerebellum. To measure the density and level of functional activation of RyR channels, we analyzed specific high-affinity binding of [^3^H]-ryanodine to membranes prepared from cerebella of males within each group. Scatchard analysis of binding data obtained from cerebellar membranes of P21 animals indicated that developmental A1254 dose-dependently increased the [^3^H]-ryanodine binding density (B_max_) without significantly changing apparent affinities (K_D_) (Figures 4A and 4B). Comparison of these values to those obtained from [^3^H]-ryanodine binding to cerebellar membranes isolated from maze-trained P31 rats indicated that maze training did not affect the density of [^3^H]-ryanodine binding sites measured in controls, but significantly increased the density of functional RyR channels among animals exposed developmentally to A1254 at 1 mg or 6 mg/kg/day, with significantly more pronounced effects observed at the higher dose (Figure 4C).

**Figure 5:**
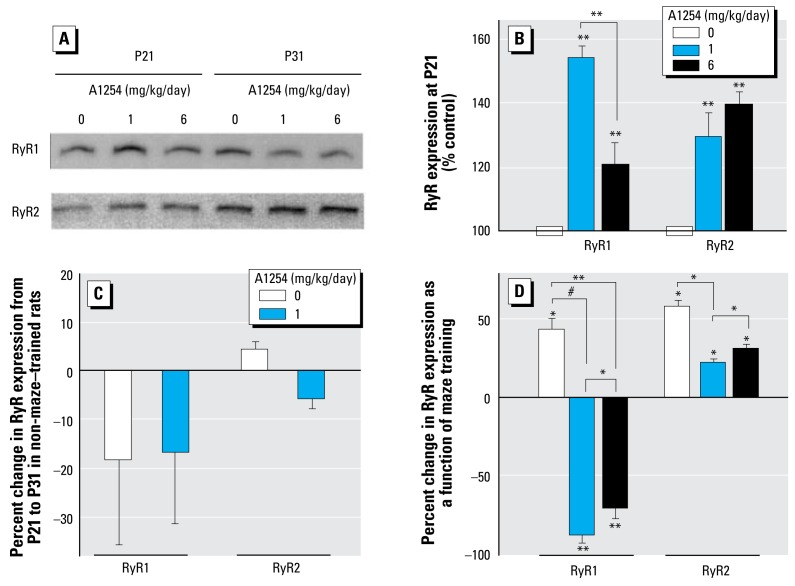
Developmental A1254 exposure alters RyR expression in the cerebellum. (*A*) Representative blot of cerebellar membrane samples from P21 and maze-trained P31 rats probed with RyR antibodies. (*B*) Densitometric analyses of RyR1 and RyR2 expression in the cerebellum at P21. Band densities of samples from A1254-treated animals are plotted as percentage of mean control band densities. (*C*) Cerebellar expression of RyR1 and RyR2 as a function of age in nonmaze-trained animals. Data are presented as percent change in mean pixel density from P21 to P31 within each treatment group. (*D*) Effects of maze training and developmental A1254 exposure on RyR1 and RyR2 expression in the cerebellum. Data are presented as mean ± SEM (*n* ≥ 4). Asterisks associated with individual bars indicate statistically significant differences between P21 and maze-trained P31 rats within a group; asterisks above horizontal lines indicate statistically significant difference between groups. *p< 0.05, **p < 0.01, *#p* < 0.001 (ANOVA; Bonferroni *post hoc*).

[^3^H]-ryanodine binds with high affinity and specificity to all three RyR isoforms and is a measure of the expression levels of functional RyR proteins as well as the stability of the open state of these channels (Buck et al. 1992). To evaluate the effects of developmental A1254 exposure on specific RyR isoforms, we examined expression levels by Western blot using monoclonal antibodies (mAbs) that selectively bind to RyR1 and RyR3 (mAb 34C) or to RyR2 (mAb C3-33) (Airey et al. 1990; Lai et al. 1992). Both RyR1 and RyR2 were observed in cerebellar membranes isolated from P21 and P31 rats, but bands corresponding to the molecular weight of RyR3 were not detected in any sample (Figure 5A). Developmental A1254 exposure increased RyR1 and RyR2 expression levels in the cerebellum of P21 rats (Figure 5B). Although the effect on RyR2 expression was similar between A1254 groups, the effect on RyR1 expression was significantly greater in the 1 mg/kg/day A1254 group relative to the 6 mg/kg/day A1254 group. Comparison of cerebellar expression levels of RyR1 and RyR2 between P21 and untrained P31 rats within controls indicated that expression of these RyR isoforms did not increase with age (Figure 5C). As previously reported for RyR2 (Cavallaro et al. 1997), maze training significantly increased RyR1 and RyR2 expression in controls (Figure 5D). In rats exposed developmentally to A1254, maze training also significantly increased RyR2 expression but significantly decreased RyR1 expression (Figure 5D). Comparison between groups of the percent change in RyR1 and RyR2 expression as a function of maze training suggested that developmental A1254 exposure reversed the effects of maze training on RyR1 expression and attenuated the effects of maze training on RyR2 expression; these responses were more pronounced in the 1 mg relative to the 6 mg/kg/day A1254 group.

### Congener-specific PCB effects on dendritic growth in cultured neocortical neurons

**Figure 6:**
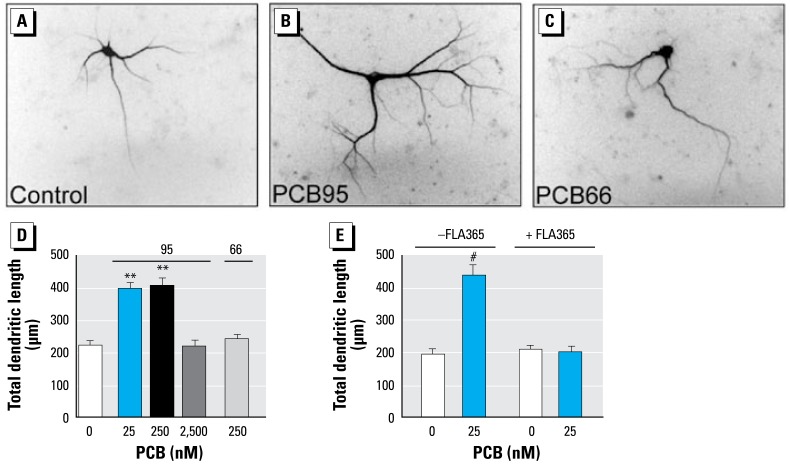
PCB95, but not PCB66, promotes dendritic growth in cultured neocortical neurons. Neurons dissociated from embryonic rat cortices were plated at high density and transfected at 6-DIV with MAP2-GFP, which labels the somatodendritic compartment of 0.5–2% of neurons in the culture. At 7-DIV, cultures were treated with vehicle (0.1% DMSO), PCB66, or PCB95. Photomicrographs of GFP-positive neurons treated with vehicle (*A*), PCB95 (*B*), or PCB66 (*C*) at 250 nM. PCB95, a congener with potent RyR activity, significantly enhanced dendritic growth in cultured cortical neurons in a nonmonotonic fashion (*B, D*). In contrast, PCB66, a congener that lacks RyR activity, had no effect on dendritic growth in cultured cortical neurons (*C, D*). (*E*) PCB95–induced dendritic growth is blocked by the ryanodine receptor antagonist FLA365 (10 μM). Data are presented as mean ± SEM (*n* = 30 neurons/condition). **p < 0.01, #*p* < 0.001 (ANOVA; Newman-Keuls post hoc).

The observation of similar inverted dose-related effects and training-dependent biphasic responses of dendritic morphology and RyR expression in A1254-exposed animals suggested a causal relationship between these effects. We previously demonstrated that noncoplanar PCBs possessing 2**–**3 chlorine *ortho* substitutions are the most potent RyR activators (Pessah et al. 2006), consistent with findings from other laboratories that noncoplanar, but not coplanar, PCBs increase intra-cellular Ca^2+^ in neurons (Kodavanti 2005). Noncoplanar PCBs at nanomolar concentrations interact with RyRs to dramatically increase their sensitivity to activation by nanomolar Ca^2+^ and attenuate their sensitivity to inhibitory feedback by millimolar Ca^2+^ and Mg^2+^ (Pessah and Wong 2001). To further probe the relationship between PCB effects on dendritic growth and RyRs, we quantified dendritic growth in primary cultures of neocortical neurons exposed to individual PCB congeners with differential effects on RyR activity at concentrations that did not adversely influence cell viability in cultured neocortical neurons (Howard et al. 2003). A 48-hr exposure of cultured neocortical neurons (7–9 DIV) to nanomolar concentrations of PCB95, a congener that potently activates RyRs (Pessah et al. 2006), significantly enhanced dendritic growth, whereas exposure to PCB66, a congener with little activity at the RyR (Pessah et al. 2006), had no effect on dendritic growth (Figure 6A–D). Interestingly, micromolar concentrations of PCB95 had no net effect on dendritic growth compared with controls, recapitulating the inverted dose-related effects of developmental A1254 exposure on dendritic growth *in vivo*. PCB-95–induced dendritic growth was completely blocked in the presence of the selective RyR antagonist FLA365 (Mack et al. 1992) (Figure 6E).

### Analyses of PCB levels in weanling rats exposed developmentally to A1254

**Figure 7:**
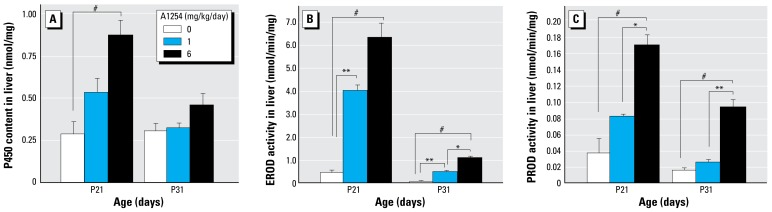
A1254 induction of CYP expression and activity in the liver is dose and age dependent. (*A*) Microsomal CYP content and (*B*) EROD or (*C*) PROD activities were measured in liver at P21 and P31. EROD and PROD were chosen as biomarkers of exposure to coplanar and noncoplanar PCBs, respectively. Liver CYP content was significantly increased relative to controls in the 6 mg/kg treatment group on P21. Developmental A1254 exposure dose-dependently increased EROD and PROD activity at P21 and P31; the increase in PROD activity was significantly different from control only in the 6 mg/kg/day A1254 group. Data are presented as mean ± SEM (*n* = 5–7/group at P21; *n* = 10–13/group at P31). *p < 0.05, **p < 0.01, #*p* < 0.001 (ANOVA; Newman-Keuls post hoc).

Our *in vitro* observations strongly suggest that noncoplanar PCB congeners mediated the effects of A1254 *in vivo*. As an indirect test of this hypothesis, we measured CYP activities in hepatic microsomes and quantified levels of individual PCB congeners in the whole brain obtained at P31 from male and female littermates of animals trained in the Morris water maze. Total CYP content was significantly increased by developmental A1254 exposure at P21, but only in the 6 mg/kg/day treatment group, and this effect was no longer evident at P31 (Figure 7A). EROD and PROD activity represent CYP isozymes differentially up-regulated by coplanar and noncoplanar PCBs, respectively (Hansen 1999). Developmental A1254 exposure dose-dependently increased EROD (Figure 7B) and PROD (Figure 7C), and these effects persisted until P31, although the absolute levels of EROD and PROD activity decreased in all treatment groups with increasing age (Figure 7C).

Of the 32 congeners chosen for analysis based on their toxicity, presence in A1254, abundance in environmental samples, and analytical capability, 30 were below the detection limit in brains of controls; the two congeners that were detected, PCB158 and PCB187, were found in only one of four samples (Table 2). In contrast, 14 congeners were detectable in brains from animals in the 1 mg/kg/day A1254 group and 16 in brains from the 6 mg/kg/day A1254 group. These were predominantly *ortho*-substituted, non-coplanar PCBs, and levels were significantly higher in the 6 mg relative to the 1 mg/kg/day A1254 group.

## Discussion

The major findings of this study are that developmental PCB exposure enhanced basal dendritic growth but decreased experience-dependent dendritic plasticity, and that these effects correlated better with altered RyR expression than with endocrine disruption.

Developmental A1254 exposure significantly enhanced dendritic growth in cerebellar Purkinje cells and neocortical pyramidal neurons among P31 rats not trained in the Morris water maze, which is consistent with our previous observations that similar exposures accelerated dendritic growth in Purkinje cells and hippocampal CA1 pyramidal neurons between P21 and P60 (Lein et al. 2007). In Purkinje cells, this effect was observed among animals in the 1 mg but not 6 mg/kg/day A1254 group, whereas in neocortical neurons, responses were comparable between A1254 groups. The reason for the different dose–response relationship in different brain regions is not known. Possibilities include regional differences in RyR regulation (Berridge 2006; De Crescenzo et al. 2006; Hertle and Yeckel 2007) or differential upregulation of cytochrome P450 enzymes by AhR ligands in the cerebellum versus neo-cortex (Iba et al. 2003), which could result in regional differences in PCB toxicodynamics and toxicokinetics, respectively.

Previous studies have shown that experience increases dendritic complexity (Greenough et al. 1979), and we observed that among controls, training in the Morris water maze significantly increased dendritic length in both Purkinje cells and neocortical neurons. However, maze training caused no change in dendritic length in the 6 mg/kg/day A1254 group and significant dendritic retraction in the 1 mg/kg/day A1254 treatment group. Structural plasticity of dendrites is considered the cellular substrate of learning and memory (Leuner and Shors 2004), and we observed that developmental A1254 exposure caused subtle but statistically significant delays in learning and memory that exhibited an inverted dose-related response similar to that observed for experience-dependent plasticity in A1254-treated animals. That these behavioral effects may be of biological significance is suggested by comparison with the human literature, which similarly demonstrates an association between developmental PCB exposures and subtle effects on cognitive function that may be overcome by training or increasing age (Carpenter 2006; Korrick and Sagiv 2008; Schantz et al. 2003). Such subtle effects may have significant biological and social costs when considered at the population level (Grandjean et al. 2007; Weiss 2000).

It is widely postulated that PCB developmental neurotoxicity is mediated by endocrine disruption (Koibuchi and Iwasaki 2006; Roman 2007; Zoeller 2007). Developmental PCB exposure is reported to modulate systemic estrogen levels (Meerts et al. 2004), increase estrogen sensitivity (Ceccatelli et al. 2006), and compete for binding to the estrogen receptor (DeCastro et al. 2006), yet we observed no A1254-related effects on plasma levels of estradiol or testosterone and only minor effects on estrogen- and androgen-dependent developmental end points. The discrepancies probably reflect differences in doses, which were generally much lower in our study, or congener profiles. As previously reported (Crofton et al. 2000; Roegge et al. 2004; Zoeller et al. 2000), developmental A1254 exposure significantly decreased serum thyroid hormone levels. However, it seems unlikely that PCB effects on dendritic growth were due to hypothyroxinemia, because *a*) neonatal hypothyroidism decreases basal dendritic growth (Ruiz-Marcos et al. 1994; Uylings et al. 1994), whereas developmental PCB exposure significantly enhanced basal dendritic growth; and *b*) PCB effects on dendritic growth were recapitulated in cultured neocortical neurons removed from systemic thyroid hormone influence. We recently demonstrated that PCB95 significantly disrupts the normal balance of excitatory and inhibitory neurotransmission in the auditory cortex at a dose that has no measurable effect on auditory brain stem responses (ABRs) (Kenet et al. 2007), which is a confirmed T_4_-dependent target of PCB (Crofton and Zoeller 2005). Although we cannot preclude the possibility that PCBs influence thyroid hormone signaling downstream of cognate receptors (Zoeller 2007), our data suggest that endocrine disruption is not the sole mechanism underlying PCB effects on neuronal connectivity. Another critical determinant of dendritic morphology is Ca^2+^ signaling (Lohmann and Wong 2005; Redmond and Ghosh 2005). Kodavanti and colleagues (Kodavanti 2005) demonstrated that noncoplanar, but not coplanar, PCBs increase intracellular Ca^2+^ in neurons. Several mechanisms have been shown to mediate this response (Kodavanti 2005; Mariussen and Fonnum 2006), but one of the most sensitive is RyR sensitization (Pessah and Wong 2001). RyR-mediated signals influence neuronal excitability, regulate synaptic plasticity (Collin et al. 2005; Conti et al. 2004; Raymond and Redman 2006; Shimuta et al. 2001), and activate cytosolic (Berridge 2006) and nuclear transcriptional events (Dolmetsch et al. 1998; Li et al. 1998) implicated in activity-dependent dendritic growth (Aizawa et al. 2004; Redmond et al. 2002; Wayman et al. 2006).

Here we show that developmental A1254 exposure dose-dependently increased RyR activity in the cerebellum as determined by [^3^H]-ryanodine receptor-binding analysis. This is most likely the result of dose-dependent accumulation of PCBs in the brain. The most abundant congeners found in the brain were noncoplanar, which are potent sensitizers of RyR channels (Pessah et al. 2006). One possible consequence of chronic RyR sensitization is altered fidelity of Ca^2+^ signaling (Marks 2002). In our study, elevated [^3^H]-ryanodine binding was closely associated with differential expression of RyR1 and RyR2 isoforms within the cerebellum, and changes in expression of isoforms were highly dependent on A1254 dose and training status. Interestingly, effects of dose and training on RyR expression closely paralleled their effects on dendritic morphology. Given the fundamental role of RyR in Ca^2+^ signaling and the critical influence of Ca^2+^ signaling on basal and activity-dependent dendritic growth, these data suggest that PCB effects on RyR could be largely responsible for the effects of developmental A1254 exposure on neuronal connectivity observed in weanling rats. In support of this hypothesis, nanomolar concentrations of PCB95, a congener that potently activates RyRs (Pessah et al. 2006), enhanced dendritic growth in primary cultures of neocortical neurons, whereas similar concentrations of PCB66, a congener with negligible effects on RyR activity, had no effect on dendritic growth. Moreover, pharmacologic antagonism of RyR activity blocked PCB95**–**enhanced dendritic growth. That PCB-mediated RyR dysfunction modulates development of neuronal networks is further supported by our previous demonstration that developmental exposure to PCB95 significantly enhanced the ratio of excitatory to inhibitory currents within the primary auditory cortex (A1) of weanling rats, which was associated with irregularly shaped topographic organization of A1 and disruption of the critical period plasticity that underlies normal postnatal auditory system development (Kenet et al. 2007). It is plausible that these effects reflect changes in basal and activity-driven dendritic complexity, as demonstrated in the present study.

Based on our findings, we propose a novel model of PCB developmental neurotoxicity in which noncoplanar PCBs sensitize RyR activity and alter Ca^2+^-dependent signaling mechanisms that link neuronal activity to dendritic growth and plasticity. A critical role for RyRs in PCB interference with neuronal connectivity suggests several explanations for the inverted dose-related effects as well as training-dependent biphasic outcomes in dendritic morphology. First, chronic RyR sensitization alters RyR expression, and consequently, downstream Ca^2+^-dependent events that regulate dendritic growth in an inverted dose-related manner, perhaps because of negative feedback at the higher A1254 dose. Second, intracellular Ca^2+^ promotes dendritic growth in a concentration-dependent manner in early neuronal development (Lohmann and Wong 2005). In later neuronal development, however, moderate increases in Ca^2+^ promote dendritic growth, whereas large increases cause dendritic retraction (Lohmann and Wong 2005; Segal et al. 2000). Thus, increasing PCB doses may increase intracellular Ca^2+^ from concentrations that promote dendritic growth to those that cause dendritic retraction. Furthermore, training-induced increases in intracellular Ca^2+^ superimposed on a background of PCB exposure may push Ca^2+^ concentrations towards those that cause dendrite retraction. Although further studies are required to test these mechanistic hypotheses, the observation of an inverted (or non-monotonic) dose–response relationship has global regulatory implications as screening and testing programs for endocrine disruption and developmental neurotoxicity that emphasize very high doses of test chemicals with little to no acknowledgement of the importance of nonmonotonic dose–response relationships are finalized (Kimmel and Makris 2001).

The relevance of our findings to human health is suggested by several considerations. First, altered dendritic growth and impaired experience-dependent dendritic plasticity are thought to contribute to the clinical manifestations of various environmentally induced neurodevelopmental disorders in humans (Connors et al. 2008; Pardo and Eberhart 2007; Zoghbi 2003). Second, congener-specific analyses of brains from weanling rats in the 1 mg/kg/day A1254 group identified predominantly *ortho*-substituted congeners at concentrations ranging from 0.5 ng to 3 ng/g wet weight. Analyses of PCB levels in human brains obtained from the general adult population similarly identified predominantly *ortho*-substituted congeners at concentrations ranging from 0.07 ng to 12 ng/g wet weight (Chu et al. 2003; Covaci et al. 2002; Dewailly et al. 1999). *Ortho*-substituted congeners with the highest activity towards RyRs, including PCB95, collectively represent 40–50% of total PCBs currently found in environmental and biotic samples, and their net effects are likely to be additive (Pessah et al. 2006). Even lower levels of PCB exposure might amplify adverse effects in genetically susceptible individuals (Campbell et al. 2006), particularly if both the genetic factor and PCBs converge to dysregulate the same developmental process. Interestingly, genes that encode Ca^2+^-regulated signaling proteins involved in synapse formation and dendritic growth are implicated in neurodevelopmental disorders (Krey and Dolmetsch 2007). Considered together, these observations identify PCBs, particularly *ortho*-substituted PCBs with high RyR activity, as candidate environmental risk factors in neurodevelopmental disorders and provide important new clues about the possible role of RyR in contributing to environmentally triggered neurodevelopmental deficits.

## Figures and Tables

**Table 1:** Effects of developmental A1254 exposure on sex steroid-dependent reproductive developmental end points in rats.

	A1254 (mg/kg/day)		
	0	1	6
Male:female ratio^a^	1.05 ± 0.12	1.07 ± 0.22	2.16 ± 0.42*
AGD (mm)^a^			
Male			
P2	3.96 ± 0.14	4.16 ± 0.25	3.93 ± 0.11
P10	7.70 ± 0.26	8.07 ± 0.36	7.76 ± 0.46
P21	16.2 ± 0.53	16.9 ± 0.59	16.9 ± 0.63
Female			
P2	1.98 ± 0.08	2.05 ± 0.19	2.08 ± 0.21
P10	5.14 ± 0.15	5.76 ± 0.21*	5.70 ± 0.27*
P21	9.48 ± 0.29	9.39 ± 0.26*	8.82 ± 0.28*
Puberty (days)^a^			
Males			
Preputial separation	40.36 ± 0.28	40.45 ± 0.14	40.21 ± 0.08
Females			
Vaginal opening	40.05 ± 0.05	40.50 ± 0.19	43.44 ± 1.07*
Adulthood^a^			
Males			
Testes^b^ (g)	2.98 ± 0.06	2.95 ± 0.11	3.11 ± 0.03
Epididymal fat pad^b^ (g)	3.66 ± 0.13	3.44 ± 0.14	3.31 ± 0.33
Epididymides^b^ (g)	0.73 ± 0.03	0.71 ± 0.02	0.69 ± 0.02
Seminal vesicles^b^ (g)	0.23 ± 0.01	0.25 ± 0.02	0.23 ± 0.01
Prostate gland^b^ (g)	0.49 ± 0.02	0.57 ± 0.03*	0.41 ± 0.01
Testosterone (ng/mL)	5.54 ± 0.87	4.53 ± 0.51	4.40 ± 0.81
Sperm count (× 10^6^)	10.40 ± 0.58	9.46 ± 1.15	11.22 ± 0.46
Females			
Ovaries^b^ (g)	0.12 ± 0.006	0.13 ± 0.009	0.12 ± 0.004
Ovarian fat pad^b^ (g)	4.19 ± 1.04	2.43 ± 0.21	2.19 ± 0.27
Uterine horns^b^ (g)	0.29 ± 0.01	0.37 ± 0.05	0.31 ± 0.05
Estradiol (pg/mL)	65.43 ± 6.34	74.80 ± 4.30	68.94 ± 3.57

a Mean ± SEM (*n* ≥ 30/group).

b Absolute organ mass.

* *p* < 0.05.

**Table 2:** Congener-specific analysis of P31 brains.

	PCB (ng/g wet weight)			PCB (ng/g lipid)		
A1254 (mg/kg/day)	0	1	6	0	1	6
Mono-*ortho*^a^						
PCB70	BDL	0.5 ± 0.2	0.6 ± 0.2	BDL	8.8 ± 2.9	11 ± 4
PCB74	BDL	2.0 ± 0.9	2.8 ± 1.3	BDL	36 ± 16	50 ± 24
PCB118	BDL	29 ± 6.1	78 ± 28 **, #	BDL	537 ± 103	1,398 ± 517*, #
PCB105	BDL	11 ± 3.0	23 ± 11*	BDL	199 ± 52	408 ± 208
PCB156	BDL	4.3 ± 1.1*	18 ± 2.0**, #	BDL	80 ± 22*	329 ± 41**, #
PCB189	BDL	BDL	0.8 ± 0.3*	BDL	BDL	14 ± 5*
Di-*ortho*^a^						
PCB99	BDL	22 ± 3.0*	83 ± 10**, #	BDL	398 ± 44*	1,479 ± 200**, #
PCB138	BDL	30 ± 1.1*	133 ± 18**, #	BDL	553 ± 18	2,369 ± 357**, #
PCB153	BDL	22 ± 0.7	110 ± 17**, #	BDL	412 ± 15	1,964 ± 336**, #
PCB128	BDL	5.3 ± 0.8*	10 ± 2.7**, #	BDL	97 ± 13*	171 ± 49**, #
PCB180	BDL	3.4 ± 0.3*	15 ± 2.0**, #	BDL	62 ± 5	275 ± 39**, #
PCB170	BDL	2.8 ± 0.2	13 ± 2.0**, #	BDL	52 ± 4	230 ± 40**, #
PCB158	1.1^b^	3.9 ± 0.2*	14 ± 2.0**, #	23.7^b^	72 ± 5	256 ± 39**, #
PCB166	BDL	BDL	1.3 ± 0.2**, #	BDL	BDL	22 ± 3**, #
Tri-*ortho*						
PCB187	1.0^b^	1.9 ± 0.3*	6.2 ± 0.7**, #	22.6^b^	35 ± 5	111 ± 14**, #
PCB183	BDL	1.0 ± 0.1*	3.7 ± 0.4**, #	BDL	19 ± 3*	66 ± 8**, #

BDL, below the detection limit.

a PCB congeners below detection limit in all samples: mono-*ortho*-substituted congeners 8, 28, 60, 66, and 114; di-*ortho*-substituted congeners 44, 49, 52, 82, 87, and 101; non-*ortho*-substituted congeners 37, 77, 126, and 169. Mean ± SEM (*n* = 4 per group).

b Concentration detected in one of four samples.

* *p* < 0.05 relative to control

** *p* < 0.01 relative to control

# *p* < 0.05 relative to 1 mg/kg/day A1254.
